# Psychologische Frauenbefreiung: Feministische Therapie zwischen Psychologie und Frauenbewegung in der Bundesrepublik der 1970er Jahre

**DOI:** 10.1007/s00048-024-00403-3

**Published:** 2024-11-14

**Authors:** Vera Luckgei

**Affiliations:** Institut für Geschichte und Ethik der Medizin, Universität Heidelberg, Heidelberg, Deutschland

**Keywords:** Feministische Therapie, Frauenberatungsstellen, Frauentherapiekongress, Akademische Psychologie, Feministische Psychologie, Feminist therapy, Women’s counseling centers, Women’s therapy congress, Academic psychology, Feminist psychology

## Abstract

Die zweite Welle der Frauenbewegung erfasste ab den späten 1960er Jahren sämtliche Bereiche der westdeutschen Gesellschaft. Dazu gehörten Debatten darüber, wie die Gesundheitsversorgung von Frauen selbstbestimmt, frauenfreundlich und feministischen Idealen entsprechend gestaltet werden könnte. Dies wurde auch in Hinblick auf die damals allgemein boomende Psychotherapie diskutiert. Der vorliegende Beitrag geht der Frage nach, wie Debatten rund um eine feministische Therapie in der Bundesrepublik aufkamen. Dabei rückt das spannungsgeladene Verhältnis zwischen Psychologie und Psychotherapie in den Blick. Während feministische Frauenberatungsstellen und Therapiezentren im deutschsprachigen Raum seit den späten 1970er Jahren ein verbreiteter Bestandteil des psychosozialen Versorgungsnetzes wurden, blieb die wissenschaftliche Psychologie feministischen Einflüssen gegenüber weitgehend unbewegt. Der Artikel zeichnet nach, wie es zu diesem Ungleichgewicht zwischen feministisch-therapeutischer Praxis und psychologischer Frauenforschung kam. Dazu setzt er im Jahr 1974 an, als Psychologinnen versuchten, feministische Impulse in die akademische Psychologie zu tragen und feministische Aktivistinnen psychotherapeutische Ansätze für die Frauenbewegung nutzbar machten. Dass viele Psychologinnen ihr Engagement aus dem akademischen in das therapeutische Feld verlagerten – so die These des Artikels –, lag an den wenig förderlichen Bedingungen, die feministisch orientierte Psychologinnen in der deutschsprachigen akademischen Psychologie vorfanden.

## Abstract (en)

From the late 1960s onwards, the early second women’s movement encompassed all areas of West German society. This included debates about how women’s healthcare could be improved in a self-determined, women-friendly way and in line with feminist ideals. These debates were also held with regard to the general boom in psychotherapy at the time. This article explores the question of how debates around feminist therapy emerged in the Federal Republic of Germany. It also looks at the tense relationship between psychology and psychotherapy. While feminist women’s counselling and therapy centers became a widespread part of a psychosocial care network from the late 1970s onwards, scientific psychology in German speaking countries remained largely closed to feminist influences. The article traces how this imbalance between feminist therapeutic practice and psychological women’s research came about. Therefore, the article sets out from 1974, when psychologists tried to introduce feminist impulses into academic psychology and feminist activists made psychotherapeutic approaches usable for the women’s movement. Many female psychologists shifted their commitment from the academic to the therapeutic field. It is argued, that this was due to the less than conducive conditions that feminist-oriented psychologists found in German-speaking academic psychology.

## Einleitung und Forschungsstand

Die sogenannte Zweite Welle der internationalen Frauenbewegung setzte in der Bundesrepublik ab den späten 1960er Jahren tradierte Geschlechterverhältnisse in Bewegung. Die Gesundheit von Frauen^1^ und ihr Recht auf körperliche Selbstbestimmung wurden zu derart prominenten Themen, dass sich daraus eine eigene Bewegung entwickelte – die Frauengesundheitsbewegung. Deren Anliegen konzentrierten sich anfangs auf den reproduktiven Bereich, doch bald griffen auch Frauen mit psychotherapeutischen Interessen die Idee der Selbsthilfe auf. Im *Frauenjahrbuch*, das jährlich über die neusten Entwicklungen der Frauenbewegung informierte, brachten Annemie Blessing et al. diese Idee folgendermaßen auf den Punkt: „So wie wir uns durch die § 218- und Selbsthilfebewegung die Macht über unseren Körper zurückholen, ebenso erobern wir uns mit der feministischen Therapie die Macht über unsere Psyche zurück.“ (Blessing et al. 1977: 199f.) Ausgehend von diesem durchaus kämpferisch formulierten Ziel galt es, eine feministische Therapie zu entwickeln.

Dieses Vorhaben wurde ab den späten 1970er Jahren umgesetzt, und zwar weitgehend abseits etablierter Psychotherapieschulen und Universitäten. Wie eine feministische Therapie aussehen sollte, welche Methoden für die Emanzipation von Frauen besonders förderlich sein könnten, auf welche Grundsätze sie sich berufen sollte und wie mit dem Aspekt der Macht innerhalb der Therapie umgegangen werden sollte – all diese Fragen wurden auf dem eigens zu diesem Zweck organisierten Frauentherapiekongress durchaus kontrovers diskutiert. Der Kongress fand von 1977 bis 2000 jährlich statt. Dass 1977 bereits ein eigener Kongress für feministische Therapie ins Leben gerufen wurde, irritiert angesichts der Tatsache, dass die akademische Psychologie zwischen 1970 und 1990 im deutschsprachigen Raum nicht den Anschein erweckte, von feministischen Positionen durchdrungen zu sein (vgl. Ruck et al. 2018; Sieben & Scholz 2012). In anderen westlichen Ländern wie den USA (Rutherford & Granek 2010), Großbritannien (Wilkinson 1990) oder Kanada (Pyke 2001) war dies durchaus der Fall.

Die Schieflage, die sich in der Bundesrepublik seit den 1970er Jahre zwischen akademischer Psychologie und feministisch-therapeutischer Praxis auftat, bildet den Spannungsbogen, den dieser Beitrag beschreibt. Er will einen Zusammenhang zwischen der ausgebliebenen Institutionalisierung feministischer Positionen in der akademischen Psychologie und der schnellen Verbreitung feministischer-therapeutischer Praxis aufzeigen. Die zentrale Forschungsfrage des Beitrags lautet daher: Wie kam es dazu, dass bereits 1977 ein Kongress für feministische Therapie ins Leben gerufen wurde, während feministische Positionen in der akademischen Psychologie weitgehend unsichtbar blieben? Anhand bisher weitgehend unbekannten Quellenmaterials wird analysiert, wie Psychologinnen versuchten, feministische Impulse in ihre Disziplin zu tragen. Erweiternd dazu wird untersucht, wie und wann die Idee feministischer Therapie in der BRD aufkam und welchen Anteil Akteurinnen aus dem Umfeld der akademischen Psychologie und der Frauenbewegung daran jeweils hatten. Um die Akteurinnengruppen in ihrem frauenpolitischen Kontext zu verorten, wird auch die Frage nach deren Verflechtungen in der internationalen Frauenbewegung beleuchtet. Zudem nimmt der Beitrag das spannungsreiche Verhältnis zwischen Psychologie und Psychotherapie in den Blick. Dieses repräsentiert, wie Martin Wieser und Lisa Malich (2021: 177) ausgedrückt haben, „einen altbekannten Zankapfel in Deutschland“.

Für die Analyse wird vorwiegend Quellenmaterial aus dem Archiv der Münchner Frauengesundheitsbewegung herangezogen, dessen Bestände sich im Stadtarchiv München (StAM) befinden (vgl. FAM 2011).^2^ Die Analyse führt ins Jahr 1974 zurück, als feministische Aktivistinnen und Psychologinnen weitgehend unabhängig voneinander feministisch-psychologische und therapeutische Impulse aus der internationalen Frauenbewegung aufgriffen und daraus eigene Programme für die Psychotherapie sowie für die akademische Psychologie entwickelten. Vor der Analyse des Materials werden drei zentrale thematische Kontexte grob skizziert: die Frauengesundheitsbewegung, die akademische Psychologie mit ihrer Fachgesellschaft sowie das in den 1970er Jahren entstehende therapeutische Feld.

### Feministinnen in Psychologie und Psychotherapie

Die Frauenbewegung mischte sich mit ihrem Kampf für die Legalisierung des Schwangerschaftsabbruchs nicht nur in die Politik, sondern auch ins Strafrecht und in die Medizin ein. Wie wichtig gesundheitspolitische und gesundheitsbezogene Selbsthilfeinitiativen für die Bewegung auch im weiteren Verlauf waren, rückt gegenwärtig vermehrt in den Fokus zeithistorischer Forschung. Das für die bundesdeutsche Bewegung richtungsweisende US-amerikanische *Women’s Health Movement* ist bereits seit Längerem Gegenstand umfassender Forschungsarbeiten (Nelson 2015; Davis 2007; Boehm 2013). Für die westdeutsche Frauengesundheitsbewegung wurden die Bereiche Sexualität und Reproduktion(-srechte) bisher am stärksten erforscht (vgl. Heinemann 2021; Stolzenberg 2000). Besonders umfassend beleuchtet Susanne Boehm in ihrer jüngst vorgelegten Publikation die theoretischen Grundlagen der Bewegung und die praktische Umsetzung am Beispiel des Berliner Frauengesundheitszentrums. Dieses Projekt der Frauengesundheitsbewegung war in seiner Frühphase ebenfalls auf medizinische und gynäkologische Hilfe und Selbsthilfe ausgerichtet (Boehm 2024).

Zu ihrer Anfangszeit war die westdeutsche Frauengesundheitsbewegung allgemein primär in Frauenprojekten organisiert, die auf Selbstorganisation von Frauen für Frauen basierten. Seit den 1970er Jahren entstanden aus Frauengruppen diverse Projekte, die sich in der Bewegung zusammengefunden hatten und ihre im aktivistischen Umfeld begonnene Arbeit professionalisieren wollten. Sie verfolgten das gemeinsame Ziel, eine vielfältige feministische Gegenkultur zu schaffen: Es wurden Buchläden, Cafés und Theater gegründet (Brückner 2019), aber auch Schutzräume für Frauen wie die Frauenhäuser (Benkel 2021) oder Archive, in denen Zeugnisse der Frauenbewegung gesammelt und archiviert wurden (Schnalzger 2023). Frauenverlage machten feministische Belletristik sowie Fachliteratur verfügbar, Frauenzeitschriften bildeten feministische Diskurse ab (vgl. Doetz 2024). Innerhalb autonomer Kollektive galten auch sogenannte Frauenraubdrucke als probates Mittel, um Texte innerhalb der Szene zugänglich zu machen.

Laut Yvonne Doderer und Beate Kortendiek (2008: 887) wurden mit Frauenprojekten Frauenräume geschaffen, die „emanzipatorische Praxis- und Handlungsfelder“ eröffnen sollten. Die feministischen Grundsätze der Frauenprojekte beinhalteten gemäß den Autorinnen folgende Punkte: „[frauen-]parteilicher und frauenzentrierter Ansatz, Arbeit von und für Frauen, Selbstbestimmung, Selbsthilfe und Selbstorganisation sowie antihierarchische und basisdemokratische Organisationsformen“ (ebd.: 889). Diese Grundsätze prägten auch die Frauengesundheitsprojekte – und zwar sowohl bei der Arbeit mit Frauen, die sie als Nutzerinnen aufsuchten, als auch bei der Zusammenarbeit im Frauenteam.

Auch die zahlreichen Frauenberatungsstellen und Therapiezentren sind diesen feministischen Projekten zuzuordnen. Die ersten solcher psychosozialen Projekte entstanden Ende der 1970er Jahre in Berlin (West)^3^ und München. Die Verankerung des Münchner Frauentherapiezentrums innerhalb der dortigen Frauenbewegung der 1970er Jahre hat Elisabeth Zellmer herausgearbeitet (2011). Anfang der 1990er Jahre gab es in Westdeutschland bereits mindestens 85 psychosoziale Frauenberatungsprojekte (Bilden 1992: 250). Dieses stark vernetzte Feld der psychosozialen Praxis bildete den feministischen Psy-Sektor (Klöppel et al.: 2024). Die Frauenberatungsstellen oder Frauentherapiezentren verfolgten das weit gefasste Ziel, Frauen in sämtlichen Lebens- und Problemlagen durch Beratung, Therapie, die Initiierung von Selbsthilfegruppen sowie durch Freizeitangebote zu unterstützen. Ihre Mitarbeiterinnen hatten neben einem akademischen Abschluss meist auch eine therapeutische Zusatzausbildung absolviert. Innerhalb der interdisziplinären Teams waren Psychologinnen eine der am stärksten vertretenen Berufsgruppen (Bilden 1992). In der praktischen Arbeit übertrugen Beraterinnen Konzepte aus der Frauenbewegung in die Beratung und Therapie.

Das wichtigste Konzept, das in dieser Frühphase der Frauenberatungsstellen vielfach in den psychosozialen Bereich übertragen wurde, war das der in den USA entwickelten *Consciousness-Raising-*Gruppen (CR-Gruppen) (Ruck 2015; Brodsky 1977). In CR-Gruppen trafen sich Frauen regelmäßig zum Gespräch. Durch den Austausch unter Frauen sollten sie erkennen, dass viele ihrer Probleme nicht – wie häufig angenommen – auf persönliche Schwächen oder Fehlleistungen zurückzuführen, sondern vielmehr strukturell bedingt waren und daher von anderen Frauen geteilt wurden. Pamela Allen, aus deren Feder eine in den 1970er Jahren verbreitete Konzeption der CR-Gruppen stammte, beschrieb den Gruppenprozess folgendermaßen:

Sie [Anm. d. Verf.: die Gruppe] ist ein Raum, in dem Frauen nicht nur zu dem Verständnis gelangen können, wie diese Gesellschaft funktioniert, um die Unterdrückung der Frauen aufrechtzuerhalten, sondern auch, wie man diese Unterdrückung psychologisch und gesellschaftlich bekämpfen und besiegen kann (Allen 1977: 59).

Männer waren hier nicht zugelassen, da Feministinnen der 1970er Jahre deren zumindest vorläufigen Ausschluss als Grundlage dafür betrachteten, um unter Frauen an sich selbst und den eigenen Positionen zu arbeiten. Das bezog sich im Übrigen nicht nur auf CR-Gruppen, sondern war in der Frauenbewegung eine generell verbreitete Praxis – auch in Frauenprojekten oder auf Frauenkongressen.

Ruck et al. (2022) haben darauf hingewiesen, dass die CR-Gruppen den deutschsprachigen Raum teilweise in einer bereits psychologisierten Form erreichten. Eine solche Variante von CR-Gruppen machte insbesondere die Psychologin Angelika C. Wagner bekannt – etwa über sozialpsychologische Fachliteratur und feministische Literatur, aber auch in Frauenzeitschriften wie der *Brigitte *(ebd.). Katharina Lux (2019) hat anhand verschiedener CR-Gruppenkonzepte herausgearbeitet, dass sich deren Zielsetzungen wandelten. So hat sie gezeigt, dass bei Varianten, in denen psychische Komponenten wie die Steigerung des Selbstvertrauens in den Vordergrund gerückt wurden, Aufrufe zum politischen Handeln wegfielen. Auch Jens Elberfeld (2020) beschäftigt sich in seiner Studie zur Therapeutisierung der westdeutschen Gesellschaft seit den 1970er Jahren am Rande mit den CR-Gruppen. Historische Studien zu feministischen Praktiken im weiteren Kontext der Psychotherapie existieren also durchaus.

Zu der Frage hingegen, wie Psychologinnen innerhalb der Disziplin feministische Impulse aufgriffen, liegen kaum Forschungsarbeiten vor. Der augenscheinlichste Grund hierfür ist, dass sich feministische Ansätze in der deutschsprachigen akademischen Psychologie kaum verankern konnten (vgl. Sieben & Scholz 2012). Einige Arbeiten zeigen jedoch, dass auch an deutschsprachigen psychologischen Instituten zeitweise feministische Inhalte vermittelt wurden. Die Psychologin Verena Lappe (1997) verdeutlicht in ihrer Studie zu Frauenseminaren, Frauenförderung und Frauenforschung am Psychologischen Institut der Universität Hamburg, dass es dort in den 1970er Jahren ein breites feministisches Lehrangebot gab. Auch am Psychologischen Institut der Universität Wien gab es seit Mitte der 1980er Jahre einen – wenn auch inoffiziellen – Studienschwerpunkt zu sogenannten frauenspezifischen Themen. Hier vermittelten Psychologinnen aus der psychosozialen Frauenberatung über 20 Jahre lang in Praxis und Selbststudium erworbene Kenntnisse über die psychosoziale Situation von Frauen (Luckgei et al. 2020). Auch am Psychologischen Institut der Freien Universität Berlin etablierte sich in den späten 1970er Jahren ein „Studien- und Arbeitsschwerpunkt ‚Feministische Wissenschaft/Psychologische Frauenforschung‘“ (Kurth 1994: 66). Die in den 1990er Jahren erhobene Forderung nach einer langfristigen Institutionalisierung durch eine der Frauenforschung gewidmete Professur wurde jedoch auch hier nicht erfüllt.

Dass sich feministisch-psychologische Ansätze in der deutschsprachigen Psychologie nicht längerfristig institutionalisieren konnten, erstaunt nicht zuletzt deshalb, da genau dies in anderen Ländern ausgesprochen erfolgreich gelang. In den USA wurde die „Psychology of Women“ 1975 offiziell als neue Teildisziplin der akademischen Psychologie vorgestellt (Brown Parlee 1975). Dort hatten Psychologinnen seit Anfang der Dekade erfolgreich feministische Impulse aus der amerikanischen Frauenbewegung aufgenommen und in die akademische Psychologie eingebracht. Als besonderes relevant wird dabei die erfolgreiche Agitation im Umfeld der American Psychological Association (APA) angesehen – dem US-amerikanischen Fachverband der Psycholog*innen. Psychologinnen, die sich an den ihrer Ansicht nach patriarchalen Verhältnissen in der APA störten, gründeten 1970 die autonome und radikalfeministisch ausgerichtete Association for Women in Psychology (Tiefer 1991). Mit der Division 35: Psychology of Women von 1973 und dem Committee on Women in Psychology 1974 folgten zwei weitere, gemäßigtere Frauenorganisationen innerhalb der APA. In der Verankerung dieser Organisationen innerhalb der offiziellen Berufsvertretung von Psycholog*innen sehen Elizabeth Johnston und Ann Johnson (2018) die Grundlage für die Entwicklung und Institutionalisierung der „Psychology of Women“. In Deutschland und der Deutschen Gesellschaft für Psychologie (DGPs) gibt es bis heute kein Pendant dazu.

### Psychologie und Psychotherapie im Wandel der 1970er Jahre

Ein Studium der Psychologie wurde im Untersuchungszeitraum allgemein und speziell bei Frauen immer beliebter. Seit den 1970er Jahren stellen sie die Mehrheit der Psychologiestudierenden.^4^ Der Anstieg des Frauenanteils in der Disziplin veranlasste Wieser und Malich zu folgender Feststellung:

In nur zwei Generationen hat sich die deutsche akademische Psychologie von einer Grundlagenwissenschaft, die von einem überschaubaren, männlich dominierten Kreis aus dem Bildungsbürgertum bespielt wurde, in eine stark auf das klinische Feld ausgerichtete, frauendominierte Profession mit bald hunderttausend von Studierenden gewandelt (Wieser & Malich 2021: 178).

Zeitgleich zum massiven Andrang der Studierenden auf das Fach Psychologie in den 1970er Jahren (vgl. Tändler 2016) verschlechtere sich die Arbeitsmarktlage für Psycholog*innen. Zwischen September 1974 und 1975 stieg deren Arbeitslosenrate um 115 Prozent (Feger 1977: 21). Von den arbeitslosen Psycholog*innen war etwas mehr als die Hälfte weiblich und knapp drei Fünftel waren „frisch [D]iplomierte[…] ohne Berufspraxis“ (ebd.).

Zudem durchlief die akademische Psychologie seit den 1970er Jahren einen Wandel hin zu einer vermehrt auf die Lehre von Psychopathologie und deren Diagnostik ausgerichteten Disziplin. Die Klinische Psychologie galt zunächst als weiblich konnotierter Teilbereich innerhalb der Psychologie (vgl. Schönpflug 2022) und an den westdeutschen Universitäten war sie bis in die 1960er Jahre ein Nischenfach gewesen (vgl. Rzesnitzek 2017). Das änderte sich, als das Fach Klinische Psychologie 1973 zum Pflichtfach im Diplomstudium wurde (vgl. Malich 2021).

Damals war die Psychotherapie seit wenigen Jahren kassenärztlich anerkannt und die zur Abrechnung berechtigten ärztlichen Psychotherapeut*innen konnten die hohe Nachfrage nicht im erforderten Ausmaß abdecken. Um Abhilfe zu schaffen, durften seit 1972 auch Psycholog*innen mit entsprechender Zusatzausbildung auf ärztliche Delegation hin therapeutische Leistungen über die Krankenkasse abrechnen. Außerdem durften Psycholog*innen als Heilpraktiker*innen therapeutisch tätig werden oder psychologische Beratung ohne therapeutischen Anspruch anbieten. Wolfgang Schneider hat gezeigt, dass der praxisnahe Bund Deutscher Psychologen diese Entwicklung begrüßte, indem er „die Heilkunde zum typischen Merkmal des Psychologenberufs erklärte“ (2024: 64), während die DGPs sich gegen die „Verheilkundlichung“ (Heckhausen 1983: 50) des Faches aussprachen.

Bis zum Inkrafttreten des Psychotherapeutengesetzes 1998 waren die Bedingungen für therapeutisch arbeitende Psycholog*innen wenig geordnet, unsicher und unübersichtlich (Schulte 2021). Die eher diffuse Ausgangslage psychologischer Berufspraxis sorgte unter anderem dafür, dass viele Psycholog*innen sich neue Tätigkeitsfelder erschlossen. Dass dies auf sehr vielfältige Weise geschah, zeigt der sogenannte Psychoboom der 1970er Jahre. Im Zuge dieser von einem „linksalternativen Milieu“ geprägten Entwicklung fanden neue, meist aus den USA stammende psychotherapeutische Methoden auch im deutschsprachigen Raum Verbreitung (Tändler 2016). Um die umfassenden, teilweise gegenläufigen Entwicklungen des Psychobooms fassen zu können, entwickelt Elberfeld (2015: 51) den Begriff des „therapeutischen Feldes“. Dieser soll „die quer zu disziplinären und institutionellen Grenzen verlaufende Entwicklung, die divergierenden und sich zum Teil widersprechenden Konzepte und Methoden sowie die konkurrierenden Akteursgruppen analytisch […] erfassen“ (ebd.). Obgleich feministische Therapie- und Beratungsprojekte zeitgleich mit den vielfältigen psychotherapeutischen Angeboten des Psychobooms aufkamen, verorteten sie sich selbst primär in feministischen Kontexten. Ihr zentrales Forum war der Frauentherapiekongress, der bis 2000 jährlich von um die 100 bis 200 Teilnehmerinnen besucht wurde (Freytag 2003). Gabriele Freytag (ebd.) hat in ihrer Untersuchung des Wandels des Selbstverständnisses der Kongressteilnehmerinnen festgestellt, dass in der Anfangsphase Frauen aus psychosozialen Projekten den Kongress dominierten. Dass Psychologinnen eine der am stärksten vertretenen Professionen in diesem Feld stellten, wurde bereits angeführt. Der folgende Abschnitt hingegen rekonstruiert die diesem Prozess vorangegangenen Bestrebungen feministischer Psychologinnen, ihre Interessen im akademischen Feld zu vertreten und verfolgt die Verschiebung dieses Engagements in den Bereich der psychosozialen Praxis.

## Die Organisation Frauen in der Psychologie (OFP)

In der OFP hatten sich Frauen organisiert, die an der Entwicklung feministischer Psychologie und Therapie interessiert waren. Auslöser war damals die Rückkehr von Angelika Wagner (damals Hochschullehrerin in Tübingen, heute Hamburg) aus den USA gewesen, die ganz begeistert von den dortigen *concious-raising-*Gruppen berichtet hatte, in denen Frauen sich in reinen Frauengruppen zusammentaten, um über ihre Erfahrungen miteinander zu reden. Ein unerhörter Vorgang damals (Rommelspacher 1998: 101).

Die Psychologin Birgit Rommelspacher war, als sie die hier zitierte Rede auf dem 20. Frauentherapiekongress hielt, Professorin an der Alice Salomon Hochschule in Berlin. In ihre Erinnerungen zur OFP hat sich besonders eingeprägt, dass Angelika C. Wagner im deutschsprachigen Raum das Konzept der CR-Gruppen bekannt machte. Wagner trug jedoch nicht nur maßgeblich zur Verbreitung der Frauengruppen bei, sie bemühte sich mit der Initiative zur OFP zudem um eine Interessenvertretung für Psychologinnen innerhalb der akademischen Psychologie. Die OFP geht auf eine Aktion auf dem 29. Kongress der Deutschen Gesellschaft für Psychologie (DGPs) im Jahr 1974 zurück. Dieser verlief nach Ansicht zweier Teilnehmerinnen – von denen eine die frisch aus den USA zurückgekehrte Wagner war (C. S. 1983)^5^ – zu sehr in den Bahnen patriarchal geordneter akademischer Wissensproduktion. Aus dem „privaten Ärger und der Frustration zweier Psychologinnen über den total von Männern dominierten Kongreß“ verfassten diese „ein Plakat, in dem sie ihre Geschlechtsgenossinnen auf das offensichtliche Missverhältnis von weiblichen Kongressteilnehmern und nahezu völliger Bedeutungslosigkeit im Wissenschaftsbetrieb hinwiesen“.^6^ Den Charakter des Kongresses beschreibt ein Protokoll des Treffens folgendermaßen:

Nachdem die Hälfte des Kongresses vorbei war, konnte man feststellen, daß 30 bis 40 % der Teilnehmer Frauen waren, daß aber von den Referenten nur ein verschwindender Bruchteil aus Frauen bestand, die dann noch überdies meist in untergeordneten Positionen (Assistentinnen) auftraten. Frauenfragen wurden auf dem Kongreß nur am Rande behandelt, und wenn […], so waren Referent und Diskutanten Männer.^7^

An der einberufenen Versammlung nahmen etwa 40 Psychologinnen teil. Zum Auftakt wurde über die in den USA neu gegründete Association for Women in Psychology (AWP) berichtet. Die AWP wurde beschrieben als eine „mitgliederstarke und sehr aktive Organisation“, die „unter anderem dafür sorgt, daß innerhalb der Psychologie über Frauenfragen gearbeitet wird, daß hierzu regelmäßig Symposien veranstaltet werden“.^8^ Bei den anwesenden Psychologinnen fielen diese Schilderungen auf fruchtbaren Boden. Müttern war die Kongressteilnahme aufgrund mangelnder Kinderbetreuung ohnehin erschwert und die anwesenden Frauen sahen laut Protokoll auf dem DGPs-Kongress kaum Möglichkeiten, ihre eigene Arbeit angemessen zu präsentieren. Es galt als eine „Erfahrungstatsache“^9^, dass auf dem Kongress nur sehr selten frauenspezifische Themen behandelt wurden. Hinzu kam, dass sich „einzelne Frauen, die an solchen Themen arbeiten, nicht recht trauen, sich in einem solchen Rahmen der Kritik auszusetzen“.^10^

Um Abhilfe zu schaffen, planten die Psychologinnen bereits bei ihrer ersten Zusammenkunft ihr weiteres gemeinsames Vorgehen. So dachten sie kurz die Idee an, nach dem Vorbild der Philosophinnen zum Jahr der Frau 1975 einen eigenen Frauenkongress auszurichten. Weitaus konkreter jedoch wurden die Pläne für den nächsten DGPs-Kongress, der 1976 in Regensburg stattfinden sollte. Die Frauen beschlossen, hierfür eine Kinderbetreuung und ein Frauensymposium zu organisieren. Bis dahin wollten die Psychologinnen eigene Netzwerke aufbauen. Um den „Informationsfluss“ untereinander zu gewährleisten, wurden für jeweils unterschiedliche Standorte sogenannte Kontaktfrauen bestimmt. Diese sollten den Informationsaustausch regeln, indem sie beispielsweise „Literaturlisten, […] Informationen über Promotionsmöglichkeiten und offene Stellen“^11^ mit Kolleginnen vor Ort teilten. Es wurde außerdem eine Mitgliederkartei geplant, die Informationen über Arbeitsschwerpunkte, Publikationen, Qualifikationen, aber auch Mitgliedschaften in psychologischen Verbänden oder Frauengruppen bündeln sollte. Die Gruppe nannte sich zunächst „Interessengemeinschaft der Psychologinnen“ und beschloss, durch Inserate in Fach- und Frauenzeitschriften auch die Öffentlichkeit über ihre Existenz zu informieren.^12^ Zukünftig sollte auch eine eigene Zeitung erscheinen.

Therapeutisches Arbeiten war bei diesem ersten Treffen zunächst kein Thema. Doch noch im selben Jahr wurde die Interessengemeinschaft der Psychologinnen auf den Kongressen der damals sehr jungen Gesellschaft für Verhaltenstherapie und der Gesellschaft für Wissenschaftliche Gesprächspsychotherapie vorgestellt, um neue Mitglieder anzuwerben. Insgesamt wurden die Adressen von rund 80 Psychologinnen zusammengetragen. Die Mitgliederlisten der OFP zeigen, dass die Mitglieder aus der Gesellschaft für Verhaltenstherapie zu zwei Fünfteln aus Studierenden bestanden und gut ein Fünftel mit Jugendlichen und Kindern arbeitete. Die Mitglieder, die auf der DGPs-Tagung zusammengekommen waren, waren zur Hälfte wissenschaftliche Mitarbeiterinnen und Dozentinnen sowie zu einem Viertel Studentinnen. Unter ihnen waren zudem zwei Professorinnen.^13^ Männer waren aus der Interessengemeinschaft ausgeschlossen. Dies wurde beschlossen, nachdem ein Mann auf einem Kongress in die Frauengruppe gekommen war und sein Interesse damit bekundet hatte, „‚minderheiten‘ [sic!] zu beobachten“^14^.

Im Februar 1975 hielt die Gruppe, die sich fortan „Organisation: Frauen in der Psychologie“ nannte, ihre konstituierende Sitzung ab. Anwesend waren dabei etwa 50 Psychologinnen aus der Bundesrepublik, Berlin und der Schweiz. In Anlehnung an die AWP, die als Vorbild für die OFP galt (vgl. C. S. 1983: 50), wurde eine Organisationsstruktur mit Regionalgruppen geschaffen (vgl. Tiefer 1991). Diese sollte dabei helfen, die Distanzen zwischen den weiträumig verteilten Psychologinnen zu überbrücken. Sechs Regionalgruppen wurden unmittelbar gegründet (Heidelberg/Mannheim, Tübingen/Reutlingen, München/Regensburg, Berlin sowie Bielefeld/Münster) und weitere geplant. Als Ziele der Organisation wurden festgehalten:

- Durchsetzung von Berufsinteressen […]
- Aufbau einer Dokumentationsstelle und Informationsaustausch über wissenschaftliche Untersuchungen und Aussagen zur Rolle der Frau
- Gemeinsame Bekämpfung psychologischer Spekulationen, die die Frauen diskriminieren
- Gemeinsamer Einsatz für vermehrte empirische Forschung über die Situation der Frau und daraus resultierende Probleme
- Entwicklung und Erprobung neuer Formen von Frauentherapie und Beratung.^15^

Diese Ziele zeigen, dass die OFP zunächst primär auf die Interessen von Psychologinnen im akademischen Bereich abzielte. Die gesonderten Protokolle der Arbeitsgruppe Forschung und des Arbeitskreises „Therapie für Frauen“ zeigen jedoch auch, dass beide Themenkomplexe bereits 1975 wichtige Themen der OFP waren.^16^ Die Arbeitsgruppe Forschung behandelte die Frage, wie „eine Dokumentation aller wissenschaftlichen Arbeiten über Frauen für unsere Organisation eingerichtet werden“^17^ könne. Sie schlug vor, eine kleine Gruppe damit zu beauftragen, „die wichtigsten Zeitschriften regelmäßig“ zu lesen und eine Kartei mit Artikeln und Kommentaren anzulegen. Alle anderen OFP-Mitglieder sollten sich ebenfalls an diesem Projekt beteiligen und interessante Artikel und Kommentare einsenden. Einzelne Fachgebiete wie „Entwicklungspsychologie, Betriebs‑, Arbeits‑, Werbe‑, Klinische, Different[ielle]“ Psychologie müssten bezüglich „Vorurteilen oder verzerrten Beurteilungen von Frauen“ überprüft und „Korrekturvorschläge“^18^ gemacht werden. Für Kriterien, „nach welchen eine kritische Bestandsaufnahme der Psychologie gemacht werden soll“, blieb laut Protokoll nicht genügend Zeit. Ganz allgemein fragten sich die Teilnehmerinnen, ob die psychologische Forschung aufgrund ihrer „Methodik, Technik und Fragestellung nicht von vornherein eine ‚männliche Forschung‘“ sei.^19^ Zwar herrschte unter den Anwesenden Einigkeit darüber, dass „die Psychologie *dringend* von dem Ballast falscher Vorstellungen über Frauen befreit werden muss“, doch ob und wie diese Aufgabe zu bewältigen sei, blieb unklar.^20^

Präziser waren da bereits die Vorstellungen des Arbeitskreises „Therapie für Frauen“. Ihm zufolge sollte es Aufgabe der OFP sein, „neue Therapie- und Beratungsformen für Frauen zu entwickeln“.^21^ Diese hätten das klare Ziel, „Frauen zu stützen, zu stabilisieren, zu Selbsthilfe und Solidarisierung anzuleiten, damit sie zur Auseinandersetzung mit ihrer Umwelt, auch/gerade Männern fähig sind“.^22^ Im Arbeitskreis verhandelten die Teilnehmenden aber auch, ob „frauenspezifische Therapie“^23^ an sich überhaupt ein berechtigtes Tätigkeitsfeld sei. Über den Ausschluss von Männern wurde durchaus kontrovers diskutiert. Einig war frau sich hingegen, dass Gruppensettings zu bevorzugen seien. Wie damit umzugehen sei, dass die Therapeutin im Therapiesetting zwangsläufig eine Machtposition einnehme, wurde ebenso debattiert wie die Frage, wie mit den Begriffen Krankheit und Therapie umzugehen sei. Eine stigmatisierende Etikettierung von Kranken, aber auch eine Individualisierung sozialer Probleme galt es zu vermeiden. Schließlich hielten die Teilnehmerinnen des Arbeitskreises fest: „Therapie muß Frauen von der individuellen Selbstbezichtigung (Versagen als Individuum) und von der (selbst-) [Anm. d. Verf.: Klammer handschriftlich nachgetragen] Definition als Kranke wegbringen“.^24^ Diese Zielsetzung zeigt eine deutliche Nähe zu den Zielen der CR-Gruppen, die ebenfalls erreichen sollten, dass Frauen die Vorstellung individueller Unzulänglichkeit überwinden.

Im Protokoll des OFP-Arbeitskreises „Therapie für Frauen“ wurden immer wieder Erfahrungsberichte und Positionen der „Berliner Frauen“ oder der „Münchnerinnen“ festgehalten. Die Berlinerinnen boten laut Protokoll zu diesem Zeitpunkt bereits im Berliner Frauenzentrum Beratungen an und von den Münchnerinnen arbeiteten zwei therapeutisch am Max-Planck-Institut. Insgesamt liest sich das Protokoll dieses Arbeitskreises wie die Dokumentation einer mit Elan geführten Auseinandersetzung, bei der durchaus widersprüchliche Ansichten aufeinanderprallten und am Ende dennoch gemeinsame Ziele formuliert wurden. Dagegen wirkt das Treffen der Forschungsgruppe weit weniger konkret. Es scheint, als sei diese von der Größe der gewählten Aufgabe überwältigt gewesen.

Im Juli 1975 erschien die einzige Ausgabe der *OFP AKTUELL: Zeitung der „Organisation: Frauen in der Psychologie“*. Auch hier traten die Berlinerinnen und die Münchnerinnen in besonderer Weise in Erscheinung.^25^ Die Münchner Gruppe habe sich seit Februar regelmäßig zweimal pro Monat getroffen und eine „Informationszentrale“ aufgebaut. Laut ihres Tätigkeitsberichts habe sich die Gruppe kritisch mit psychologischer Forschung auseinandergesetzt, ein praktisches Beratungsprojekt gestartet und sich in einer Selbsterfahrungsgruppe ausgetauscht. Es war auch die Münchner Regionalgruppe, die die *OFP AKTUELL* herausgab.^26^

Aus Berlin erschien in der *OFP AKTUELL* ein Bericht der „Frauen im Gesundheitswesen“. Er war von (angehenden) Psychologinnen verfasst worden, die sich auf dem Kongress für Verhaltenstherapie zusammengeschlossen hatten.^27^ Hier wurde dargestellt, wie die Berlinerinnen die Idee der OFP ins Frauenzentrum trugen. Die Berlinerinnen hätten im Zentrum über das Treffen der OFP berichtet, daraufhin hätten sich ihnen spontan Frauen aus ganz unterschiedlichen Bereichen des Gesundheitswesens angeschlossen. Aufgrund verschiedener Interessen habe sich dieser Zusammenschluss jedoch nicht als tragfähig erwiesen und bald waren Psychologinnen und Psychologiestudentinnen unter sich. Angetrieben von dem Wunsch, ein eigenes praktisches Projekt ins Leben zu rufen, habe sich die Berliner Regionalgruppe der OFP dann mit einer Gruppe vernetzt, die im Frauenzentrum bereits Beratung anbot. Dabei blieb zunächst offen, welche organisatorischen Schritte unternommen werden müssten (Vereinsgründung, Finanzierung), welche therapeutischen Angebote für welche Zielgruppen das Projekt anbieten wollte („Frauen in der Psychiatrie, Frauen mit psychologischen Problemen“) und welche Qualifikationen die zukünftigen Beraterinnen mitbringen sollten. Fest stand hingegen, dass mit „einem eigenen Laden“ konkrete Berufsperspektiven für die beteiligten Psychologinnen (in spe) geschaffen werden sollten.^28^

In den Protokollen der konstituierenden Sitzung der OFP und der *OFP AKTUELL* zeichnete sich ein steigendes Interesse an der therapeutischen und beratenden Praxis ab. Zeitgleich wurde jedoch auch der Plan weiterverfolgt, sich als Frauenorganisation im Kontext der akademischen Psychologie zu positionieren. Für den DGPs-Kongress 1976 in Regensburg bereitete die Reutlinger Regionalgruppe der OFP, der auch Angelika C. Wagner angehörte, „ein eigenständiges ‚Frauenprogramm‘ vor mit einem eigenen Raum für die Dauer des Kongresses, regionalen Themenbeiträgen über Frauenprojekte in der Psychologie (z. B. Frauentherapie, Geschiedene Frauen, Geschlechtsspezifische Problematik u. ä.), Möglichkeiten zu informellen Treffen und gegenseitiger Information“ (C. S. 1983: 108). Was dann tatsächlich während des Kongresses im Frauenprogramm passierte, wurde allerdings nicht dokumentiert. Im Kongressbericht (Deutsche Gesellschaft für Psychologie 1977) finden sich keine Hinweise auf ein solches Programm. Somit trugen die Aktivitäten der OFP nicht zu einer langfristigen Sichtbarkeit der Frauenforschung in der akademischen Psychologie bei, sondern bewirkten auf lange Sicht sogar das Gegenteil: Wie Helga und Lothar Sprung (1996: 214f.) in einer bibliometrischen Analyse gezeigt haben, sank der Anteil von Frauen an den Vortragenden beim DGPs-Kongress von ca. 25 Prozent im Jahr 1974 auf ca. 15 Prozent im Jahr 1976. Aus der Analyse geht allerdings nicht hervor, ob die gezählten Frauen tatsächlich auf dem Kongress vortrugen oder lediglich als Co-Autor*innen genannt wurden. Ungeachtet dessen führe ich den Einbruch der Sichtbarkeit von Frauen in der Kongressdokumentation von 1976 darauf zurück, dass die Frauen, die im gesonderten Symposium vortrugen, in der offiziellen Dokumentation nicht aufschienen.

Die Psychologinnen der OFP taten sich in einem eigenen Frauenraum zusammen und Männern wurde der Zutritt zur OFP verwehrt. Mit dieser Abspaltung dürften sie den Kräften in die Hände gespielt haben, in deren Interesse es lag, dass die Aktivitäten der feministischen Organisation innerhalb der akademischen Psychologie möglichst unsichtbar blieben. Die Tatsache, dass die Anliegen der OFP nicht in der offiziellen Kongressdokumentation auftauchen, korrespondierte mit der vom DGPs-Vorstand vertretenen Auffassung, politische Themen sollten möglichst nicht in die akademische Psychologie getragen werden. Der damalige DGPs-Vorsitzende, Hubert Feger, unterstrich diese Position in seiner Rede zur Lage der Psychologie auf dem Kongress. Er betonte, dass die Entwicklung der akademischen Psychologie „allein nach wissenschaftlicher Notwendigkeit“ (1977: 15) zu gestalten sei. Externe, politisch motivierte Einflüsse – sei es in der Vergangenheit durch „die nationalsozialistische Diktatur“ oder zeitgenössisch durch staatliche Einflussnahmen oder „die Studentenrevolte“ – betrachtete Feger allesamt als Gefahren für die Freiheit der Wissenschaft (ebd.: 15). Obgleich über Fegers Position zu den Bemühungen der OFP im Speziellen oder der Frauenbewegung im Allgemeinen nichts bekannt ist, lassen seine Äußerungen darauf schließen, dass er auch dieser Form politischer Einflussnahme zumindest skeptisch gegenübergestanden haben dürfte. Mit seiner prominenten Rede bestärkte Feger seine vermeintlich unpolitische Position als Teil der hegemonialen Ordnung innerhalb der damaligen Fachgesellschaft. Die Fachgesellschaft wiederum hatte erheblichen Einfluss auf die weitere Entwicklung der Disziplin, da „sich enge Verbindungen zwischen der Genese der Psychologie in Deutschland und ihrer wissenschaftlichen Gesellschaften“ zeigen (Guski-Leinwand 2007). Susanne Guski-Leinwand und Carl Graumann haben für die deutsche akademische „Psychologie, ihre Gesellschaft und ihre Öffentlichkeiten“ Folgendes festgestellt:

[Es] wacht die (in der Regel in einer „Gesellschaft“ organisierte) Gemeinschaft der Wissenschaftler […] „intern“ über die Wissenschaftlichkeit der Arbeit ihrer Mitglieder […]. Gleichrangige, vor allem aber hochrangige und im Idealfall als Experten ausgewiesene Mitglieder der Wissenschaftsgemeinschaft urteilen, oft verbindlich, über die Arbeit eines anderen Mitglieds (Graumann & Guski-Leinwand 2004: 63).

Demzufolge ist eine Fachgesellschaft ein mächtiger Zusammenschluss, was die Entwicklung der entsprechenden Disziplin angeht. Für den konkreten Fall der OFP kommt hinzu, dass Psychologinnen, die innerhalb der Fachgesellschaft die Anliegen und Interessen von Frauen hätten aufgreifen und vertreten können, in der DGPs in den 1970er Jahren massiv unterrepräsentiert waren (vgl. Traxel 1985) und keinerlei Machtpositionen innerhalb des Vorstands bekleideten (vgl. Schneider 2024). Andere Geschlechterverhältnisse innerhalb der Fachgesellschaft hätten eine intergenerationale Zusammenarbeit junger, feministisch orientierter Psychologinnen und etablierter Psychologinnen ermöglicht. Solche Kooperationen haben Johnston und Johnson in ihrer Analyse des Prozesses der Verankerung der Frauensektion innerhalb der American Psychological Association als wichtigen Faktor benannt (2018: 16).

Nach dem DPGs-Kongress 1976 verfolgten die Psychologinnen der OFP das kollektive Ziel, die Interessen von Psychologinnen im akademischen Feld zu vertreten, nicht weiter. Auf dem Kongress wurde jedoch bereits angedacht, „workshops für Frauentherapie abzuhalten“ (C. S. 1983: 108). Die neueren Mitglieder, die auf den Kongressen der Verhaltens- und Gesprächstherapie gewonnen wurden, dürften – neben der völligen Unsichtbarkeit innerhalb der DGPs – dazu beigetragen haben, dass sich die Ausrichtung der OFP zugunsten des therapeutischen Arbeitens wandelte. Unter den Mitgliedern waren wenige im akademischen Feld etablierte Psychologinnen, hingegen viele Studentinnen und Nachwuchswissenschaftlerinnen. Diese dürften sich angesichts der damals angespannten Arbeitsmarktlage aktiv nach Arbeitsplätzen umgesehen haben. Am Beispiel der Berliner Regionalgruppe hat sich bereits angedeutet, dass auch frauenbewegte Kontexte hierbei eine Rolle spielten. Der kommende Abschnitt analysiert diesen Aspekt, indem die parallel zur Organisationsgeschichte der OFP verlaufenden Debatten und Ereignisse rund um feministische Therapie innerhalb der autonomen Frauenbewegung ins Zentrum gerückt werden.

## Anfänge der feministischen Therapie

Die Idee einer feministischen Therapie, die in der OFP massiv an Bedeutung gewann, wurde in der BRD initial in Kreisen der internationalen autonomen Frauenbewegung diskutiert. Während die Psychologinnen der OFP 1974 den Gedanken hegten, das von den Vereinten Nationen für 1975 ausgerufene „Jahr der Frau“ für einen psychologischen Frauenkongress zu nutzen, machten autonome Feministinnen international dagegen mobil. Sie befürchteten eine Vereinnahmung frauenpolitischer Anliegen durch bürgerliche Kräfte. Im bundesdeutschen Kuratorium zum Jahr der Frau unter Vorsitz der Bundesministerin für Jugend, Familie und Gesundheit, Katharina Focke (SPD), sahen sich die autonomen Feministinnen nicht angemessen repräsentiert. Die autonome feministische Bewegung wollte daher dem staatlich verordneten Programm zum Jahr der Frau ein international koordiniertes „Aktionsprogramm“ entgegenstellen (vgl. Anonym 1975: 7). Dafür wurde vom 15. bis 17. November 1974 in Frankfurt am Main ein internationaler Frauenkongress einberufen, an dem etwa 500 Frauen aus 20 Ländern teilnahmen (ebd.). Organisiert wurde der Kongress vom ersten autonomen Frankfurter Frauenzentrum, dem Frauenzentrum Eckenheim (Biblioteche Civiche Padova o. J. [1974]). Zur Entwicklung eines Gegenprogramms bildeten die Teilnehmerinnen Kleingruppen, die zu verschiedenen Themenschwerpunkten arbeiteten und die Ergebnisse ihrer Arbeit in Form von Resolutionen niederschrieben. Diese wurden dann im Kongressplenum besprochen und sollten anschließend an politische Entscheidungsträger*innen versandt werden. Dabei entstand mit der Resolution feministische Therapie die frühestens datierte und publizierte Quelle in deutscher Sprache zur feministischen Therapie.

### Resolution zur feministischen Therapie

**Abb. 1:**
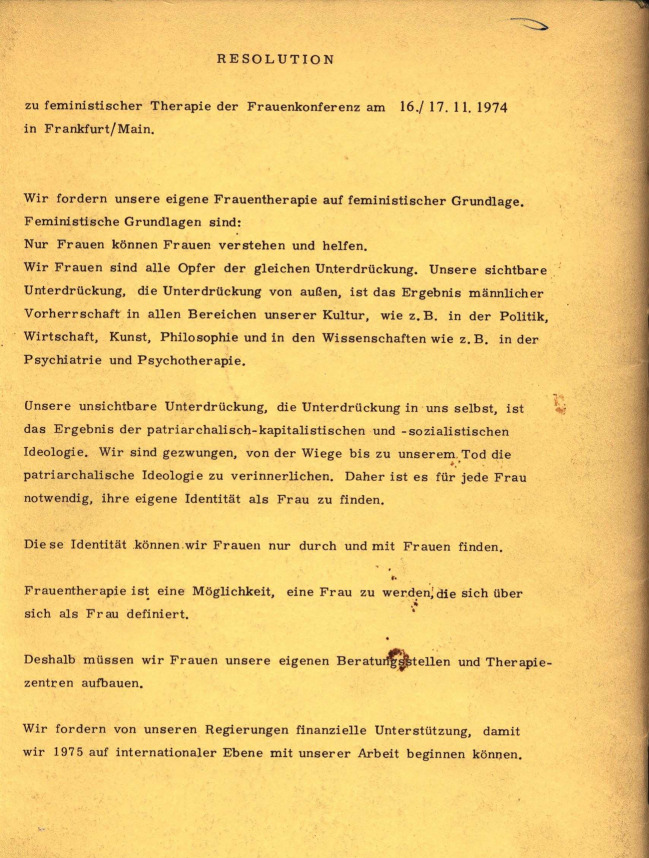
BIFF 1975: Rückseite

Der Text der Resolution (Abb. 1,^29^) macht keine Angaben über seine Autorinnen. Er ist durchgehend in der ersten Person Plural verfasst. Als Ziel benennt das anonyme Kollektiv, die durch die patriarchalen Verhältnisse bedingte „innere Unterdrückung“ zu überwinden, um eine Frauenidentität zu entwickeln, die nicht über oder von Männern, sondern im Frauenkollektiv definiert wird. Um dieses Ziel erreichen zu können, sollte in Frauenberatungsstellen und Therapiezentren eine feministische Therapie entwickelt werden. Regierungen wurden dazu aufgefordert, die praktische Umsetzung dieser Ziele durch finanzielle Unterstützung zu ermöglichen.

Die Resolution folgt einer für die frühe Zweite Frauenbewegung typischen Argumentationsstrategie, wonach alle Frauen gleichermaßen von patriarchaler Unterdrückung betroffen seien. Differenzen zwischen Frauen oder ihren jeweiligen Lebensumständen wurden als weitgehend nebensächlich betrachtet. Dies bringt die Resolution in ihrer Gleichsetzung zwischen „patriarchalisch-kapitalistische[r] und -sozialistische[r] Ideologie“ (Abb. 1) besonders einprägsam zum Ausdruck. Die ausschließliche Fokussierung auf die Differenzkategorie Geschlecht ist laut Laura Brown (2018) typisch für die frühe feministische Therapie. Im Umkehrschluss wurde Männern in der Resolution qua Geschlecht die Kompetenz für die Therapie von Frauen abgesprochen. Diese Zurückweisung männlicher Kompetenzen wurde auch auf die unter „männlicher Vorherrschaft“ entwickelten Methoden von Psychiatrie und Psychotherapie ausgeweitet. Die feministische Therapie und mit ihr die frauenbewegte Therapeutin wurden damit zu den einzigen Akteurinnen hochstilisiert, von denen im therapeutischen Feld ein glaubwürdiges Heilversprechen zu erwarten sei. Die Resolution kann daher als durchaus potente Kampfansage gegenüber der in den 1970er Jahren noch vorherrschenden männlichen Dominanz in den Reihen der Psychotherapeut*innen sowie als Distanzierung vom Psychoboom angesehen werden, der im linksalternativen Milieu florierte, allerdings keine speziellen Angebote für Frauen machte.

In der Resolution finden sich bereits die wichtigsten Grundideen feministischer Therapie. Die Postulate, nur Frauen könnten Frauen helfen und feministische Therapie müsse von Frauen entwickelt werden, beruhen auf dem Grundsatz „von Frauen für Frauen“ – in Theorie und Praxis. Diese Prämisse von Selbsthilfe und Selbstorganisation von Frauen war einer der Grundpfeiler feministischer Frauenprojekte. Feministische Therapie sollte in ebenfalls selbstorganisierten Frauenprojekten entwickelt werden – in Frauenberatungsstellen und Therapiezentren. Dort sollte wiederum Hilfe zur Selbsthilfe geleistet werden. Die Resolution postuliert des Weiteren, dass alle Frauen Opfer derselben, nämlich der patriarchalen Unterdrückung seien. Auf diese Prämisse reagierten Frauenprojekte mit der Forderung nach Parteilichkeit für Frauen: „[D]as Engagement in der Arbeit mit Frauen [wurde] von der Gewissheit getragen, bei allem, was einer Frau passiert, potentiell selbst betroffen sein zu können.“ (Scheffler 1986: 31) Die Parteilichkeit für Frauen ist ein weiterer Grundpfeiler psychosozialer und therapeutischer Arbeit mit Frauen. Im Postulat von der „unsichtbaren Unterdrückung“, der „Unterdrückung in uns selbst“ (Abb. 1) steckt die Annahme, dass Frauen patriarchale Normen verinnerlichen und unter diesen leiden. Eine weitere Prämisse der feministischen Therapie ist, dass es patriarchale Verhältnisse sind, die das psychische Leiden vieler Frauen bedingen. Die Tatsache, dass die Resolution Krankheit überhaupt nicht thematisiert, entspricht ebenfalls frühen feministisch-therapeutischen Positionen, die Krankheitsbegriffe und Diagnosen weitgehend ablehnten (vgl. Freytag 1992). Hinter dem postulierten Ziel, im Frauenkollektiv eine eigene Identität als Frau zu entwickeln, steht der Wunsch nach dem, was Ulrike Klöppel als relationale Autonomie bezeichnet hat – der größeren Unabhängigkeit von Männern und der stärkeren Bezogenheit auf andere Frauen (im Druck).

Die Resolution beinhaltete die Kernelemente der feministischen Therapie, noch bevor in der Bundesrepublik die diesbezüglichen Debatten in Gang gekommen waren. Dazu gehören die Parteilichkeit für Frauen, der Wunsch nach der Unabhängigkeit von Männern und den von ihnen entwickelten therapeutischen Methoden sowie die Zurückhaltung in Hinblick auf Krankheitsdiagnosen. Diese Grundannahmen wurden in erster Linie von den Idealen der Frauenbewegung geprägt, während Therapeuten und therapeutischem Fachwissen mit größter Skepsis begegnet wurde.

## Psychosoziale Initiative für Frauen

Die auf dem internationalen Frauenkongress angestoßene Debatte hatte weitreichende Folgen für das therapeutische Feld. Wie eingangs erwähnt, entstanden in den späten 1970er Jahren – wie in der Resolution gefordert – in allen größeren westdeutschen Städten Frauenberatungs- und Therapiezentren. Den Anfang machten einige Berlinerinnen, die am internationalen Frauenkongresses 1974 in Frankfurt am Main teilgenommen hatten. Die Gruppe BIFF: Beratung und Information für Frauen bot 1975 im Frauenzentrum Berlin erstmals psychosoziale Frauenberatung an. Neben psychologischer Beratung warb die BIFF (1975: 11) in einer Annonce anfangs auch mit „Rechtsberatung ([für] ledige Mütter)“, „Ausbildungsberatung (Berufsberatung f[ür] Mädchen)“ und „Mietberatung“. Die Beraterinnen der BIFF stellten allerdings „in ihrer Beratungstätigkeit fest […], daß eine einmalige oder mehrmalige Beratung nicht ausreicht und die Frauen für eine Veränderung ihrer psychischen oder sozialen Lage eine feste, kontinuierlich stattfindende Gruppe benötigen“ (PSIFF o. J.: 1).

An diesem Punkt im Jahr 1976 schlossen sich die Beraterinnen der BIFF mit den Psychologinnen der Berliner OFP-Regionalgruppe „Frauen im Gesundheitswesen“ zusammen. Gemeinsam gründeten sie den neuen Verein PSIFF: Psychosoziale Initiative für Frauen. In einer Selbstauskunft beschreibt die PSIFF das Motiv für den Zusammenschluss wie folgt: „Das gemeinsame Interesse unserer beiden Gruppen war, für Frauen, die ihre psychischen und sozialen Probleme nicht mehr allein in oder mit ihrer Umwelt lösen können, eine Gruppentherapie zu entwickeln und durchzuführen.“ (PSIFF o. J.: 1) Das Team der PSIFF war multidisziplinär, bestand jedoch „vorwiegend aus Diplompsychologinnen“ (Kavemann 1984: 24). Während die BIFF von neuen Aktivistinnen des Berliner Frauenzentrums übernommen wurde (BIFF 1977), widmeten sich die Frauen der neu gegründeten PSIFF dezidiert der Entwicklung gruppentherapeutischer Verfahren für Frauen. In der Anfangsphase beriefen sich die PSIFF-Beraterinnen dafür auf das Konzept der *problemsolving groups for women* von Hogie Wyckoff (PISFF o. J.) Wyckoff selbst war eine US-amerikanische Vertreterin der radikalen Psychiatrie (vgl. Richert 2019). In ihrem Therapiekonzept für Frauen verband sie Elemente der Transaktionsanalyse, die den humanistischen Therapien zugeordnet wird, mit der Idee der CR-Gruppen. In den Wyckoff’schen Problemlösungsgruppen der PSIFF sollten acht Frauen ein Dreivierteljahr lang gemeinsam an ihren Problemen arbeiten (Strödel 1979: 334). Die Gruppen wurden jeweils von einer der zehn PSIFF-Mitarbeiterinnen betreut. Im deutschsprachigen Raum machte eine von der BIFF herausgegebene Broschüre mit dem klingenden Titel *Anfänge einer feministischen Therapie* (1975) das Konzept bekannt. Diese enthielt neben der deutschen Übersetzung des Beitrags von Wyckoff ein Vorwort der Berliner Herausgeberinnen, einige Werbeannoncen autonomer Frauenberatungsstellen in Berlin sowie auf der Rückseite die besprochene Resolution von 1974. Die im Selbstverlag vier Mal aufgelegte und in der Bundesrepublik vom Frauenverlag Frauenoffensive vertriebene Broschüre fand in feministischen Kreisen in den späten 1970er Jahren viel Beachtung. Beispielsweise zogen Frauengruppen in autonomen Zentren sie heran, die in Selbsthilfegruppen Frauentherapie machen wollten (vgl. Traude 1977), und auf dem ersten Frauentherapiekongress 1977 wurde die Broschüre diskutiert.

Dass die BIFF und die Frauen der Berliner Regionalgruppe der OFP im Berliner Frauenzentrum zueinanderfanden, war durchaus nichts Ungewöhnliches. Diverse frühe Frauenprojekte entstanden durch Vernetzung und Kooperation im Frauenzentren. In einem Interview mit Cristina Perincioli beschreibt Monika Schmid, eine Mitbegründerin zuerst der BIFF und später der PSIFF, die Bedeutung von Frauenzusammenhängen für diese Projekte:

Daraus entstand ein Netz von Freundschaften, weil wir uns sehr gut kannten, uns aufeinander verlassen konnten, wo dieses von Frauen für Frauen entstand, wo wir uns ernst nahmen […]. [D]u konntest alles in die Gruppe einbringen und diskutieren lassen. Daraus entstanden Projekte, ich habe ja mindestens fünf Vereine mitgegründet. Der erste war Beratung und Information für Frauen (BIFF), der zweite war psychosoziale Initiative für Frauen (PSIFF), hier waren mehr die Psychologinnen, die sich damit ihren Arbeitsplatz schufen (Perincioli 2015: 95).

Schmid beschreibt die Atmosphäre in den Frauenzusammenhängen als wohlwollend. Dort konnten die Frauen Ideen ausprobieren und durchdeklinieren. Solche Möglichkeiten zu eröffnen gehörte zum Grundgedanken der Frauenprojekte. Hier wurden die Ideale Selbstbestimmung und Selbsthilfe sowie eine antihierarchische Einstellung hochgehalten. Damit boten diese Zusammenhänge Frauen in den 1970er Jahren eine bis dahin unbekannte Unterstützungsstruktur für die Initiierung eigener Projekte. Der Frauenraum sollte die Autonomie von Frauen fördern und bot Frauen die Möglichkeit, sich untereinander zu vernetzen, sich gegenseitig zu unterstützen und voneinander zu lernen. Schmid und ihren Mitstreiterinnen ermöglichten diese feministischen Zusammenhänge die Gründung der Vereine BIFF und PSIFF. In der PSIFF waren es laut Schmid primär Psychologinnen, die sich angesichts einer angespannten Arbeitsmarktlage ihre eigenen Arbeitsplätze schufen. Solche Projekte blieben nicht auf Berlin begrenzt. OFP und BIFF trieben die Idee der feministischen Therapie innerhalb der BRD weiter voran, wie das Beispiel des nationalen Frauenkongresses von 1977 zeigt.

## Der Weg zu einem eigenen Kongress für Frauentherapie

Die zunächst im akademisch-psychologischen Bereich verorteten Psychologinnen der OFP verlagerten ihr Engagement ab 1977 auch in autonome feministische Strukturen. Dies zeigt die Dokumentation des nationalen Frauenkongresses 1977 in München (*Frauenoffensive Extrajournal*: 1977). Initial war der Kongress geplant worden, um auf nationaler Ebene Strategien auszuarbeiten, mit denen der zunehmenden Kriminalisierung von Frauenzentren begegnet werden könnte. In der Dokumentation des Frauenkongresses nimmt die Darstellung therapeutischer Initiativen und der diesbezüglichen Debatten allerdings den prominentesten und den umfassendsten Platz ein. Neben einer Selbsterfahrungsgruppe aus Oldenburg stellten die Berliner BIFF und die „Münchner Frauen“ aus dem Umfeld der OFP ihre Überlegungen zur feministischen Therapie vor. Einem Protokoll der Münchner Frauen (1977: 8) zufolge stieß dies auf so großes Interesse, dass sich die Zuhörenden auf mehrere Räume verteilen mussten. Das Publikum war dem Protokoll zufolge „sehr heterogen“ und bestand aus „Frauen aus der Frauenbewegung und Frauen, die nicht in der Frauenbewegung sind, Therapeutinnen, die ihre Erfahrungen austauschen, Frauen, die feministische Therapie innerhalb der Frauenbewegung machen und Frauen, die selber an solchen Gruppen teilnehmen wollen“ (ebd.). Allein in München habe es im März 1977 bereits sieben Gruppen gegeben, die sich als „Psycho-Selbsthilfegruppen“ (ebd.: 4) bezeichneten. Das Münchner Modell der Psycho-Selbsthilfegruppen wurde von seinen Entwicklerinnen folgendermaßen vorgestellt:

Die Psycho-Selbsthilfegruppen sind unsere Alternative zur herkömmlichen psychotherapeutischen Praxis, die wir als sexistisch erlebt haben und ablehnen. Wir wollen gegen deren undurchschaubare Machtmechanismen unsere Eigenverantwortlichkeit für Veränderung setzen.^30^

In diesem radikal formulierten Konzept wiesen die Münchnerinnen bestehende therapeutische Ansätze aufgrund ihrer undurchschaubaren Machtmechanismen zurück. 1978 veröffentlichte die „Psychologinnengruppe München“ (1978) in einem Sammelband der Psychologen Heiner Keupp und Manfred Zaumseil eine weiterentwickelte und weniger radikal formulierte Ausführung dieses Selbsthilfekonzepts. Während sie hier auch ihre Berufsbezeichnung Psychologinnen durchaus angaben, verzichteten die Münchner Frauen auf dem nationalen Frauenkongress 1977 darauf. Den Protagonistinnen der feministischen Therapie war durchaus bewusst, dass das Projekt feministische Therapie innerhalb der Frauenbewegung nicht als frei von Widersprüchen galt. Im aktivistischen Kontext der 1970er Jahre wurde insbesondere das Machtgefälle in der hierarchisch strukturierten Therapiesituation problematisiert. Die Ungleichheit zwischen professioneller Therapeutin und hilfsbedürftiger Klientin wurde als Widerspruch zum Grundsatz der Gleichheit unter Frauen sowie zur anzustrebenden Autonomie von Frauen interpretiert. Dennoch ließ sich auch die Entwicklung einer feministischen Therapie im Sinne der Grundsätze der Frauenbewegung als Notwendigkeit ableiten:

Wir haben das Problem, daß es genug Frauen gibt, die aufgrund ihrer Situation mit ihren Problemen nicht fertig werden, Hilfe brauchen und denen frau, wenn sie ins Frauenzentrum kommt, durch alternative Formen von Therapie oder anderen Möglichkeiten helfen muß (Münchener Frauen 1977: 8).

Die Protokollantinnen empfanden es der Formulierung nach zu urteilen als ihre frauenpolitische Pflicht, therapeutische Angebote speziell für Frauen zu entwickeln. Die Anrufung der Frauensolidarität ermöglichte es den (angehenden) Therapeutinnen, sich innerhalb des Spannungsfeldes zwischen Bewegung und Profession als solidarische Fachfrauen mit feministischen Grundsätzen zu positionieren. Um diese Position zu legitimieren und zu festigen, bedurfte es allerdings weiterer Fundierung. Auch um die Debatten über diese Widersprüche möglichst produktiv führen zu können, wurde zu einem Workshop über feministische Therapie eingeladen, der zwei Monate später in Köln stattfinden sollte. Hier sollte intensiv an der Weiterentwicklung der feministischen Therapie gearbeitet werden. Wer mitmachen wollte, sollte sich anhand der vorab bereitgestellten Materialen vorbereiten, zu denen die Gruppenkonzepte der Münchner und der Berliner Frauen gehörten. Dieser Workshop wurde zum Auftakt des bis 2000 jährlich veranstalteten Frauentherapiekongresses.

## Fazit

Die Entwicklung der feministischen Therapie ging in der Bundesrepublik einher mit der Entstehung von Frauenberatungsstellen und Frauentherapiezentren sowie dem Frauentherapiekongress. Impulse für diese Entwicklungen kamen von zwei Frauengruppen, die sich relativ zeitgleich im akademischen sowie im aktivistischen Kontext formierten. Aus der internationalen Frauenbewegung wurde die Idee einer feministischen Therapie aufgegriffen und schnell in die Praxis übertragen. Erfahrungen und Konzepte wurden über die Netzwerke und Strukturen der Frauenbewegung distribuiert.

Feministisch orientierte Psychologinnen vernetzten sich in der Organisation: Frauen in der Psychologie zunächst im Umfeld der akademischen Psychologie. Doch auch hier gewann das Thema feministische Therapie schnell an Bedeutung. Dieser Prozess dürfte dadurch beschleunigt worden sein, dass die Klinische Psychologie in die Studienpläne integriert wurde, und durch einen generellen Boom im Bereich der Psychotherapie in den 1970er Jahren. Auch dürften die neuen Mitglieder der auf den Kongressen der damals neu aufgekommenen Verhaltenstherapie und der wissenschaftlichen Gesprächspsychotherapie die Ausrichtung der Organisation beeinflusst haben. Doch hatten die Psychologinnen, die in der akademischen Psychologie tätig waren, hier ebenfalls oftmals – wie sie es selbst ausdrückten – lediglich „untergeordnete Positionen“^31^ inne. Viele waren als Assistentinnen oder wissenschaftliche Mitarbeiterinnen in wenig gefestigten Anstellungsverhältnissen, die Frauen in den 1970er Jahren deutlich weniger Aufstiegschancen versprachen als ihren männlichen Kollegen. Unter den Mitgliedern der OFP fanden sich nur zwei Professorinnen. Die Gruppe, die sich besonders stark für die OFP begeisterte, bestand aus Studentinnen der Psychologie. Diese sahen sich als angehende Berufseinsteigerinnen mit einer schlechten Arbeitsmarktlage konfrontiert. Die Arbeit an und in einem selbst organisierten Frauenprojekt bot ihnen eine attraktive Alternative. Dass viele Psychologinnen und Studentinnen der Psychologie, die sich zunächst für die Interessen von Frauen in der akademischen Psychologie eingesetzt hatten, ihr Engagement zur therapeutischen Praxis im selbstorganisierten Frauenprojekt hin verlagerten, lag also nicht zuletzt an den unterschiedlichen Rahmenbedingungen beider Felder. Auf der einen Seite stand eine stark hierarchische akademische Disziplin, deren etablierte Akteur*innen als Gatekeeper fungierten. Politisch motivierte Wissenschaft galt in weiten Teilen der deutschsprachigen akademischen Psychologie der 1970er Jahre als ideologisch und fand hier wenig Anerkennung. Doch auch Forschungsrichtungen ergeben sich nicht unmittelbar aus der Wissenschaft selbst – oder wie der DGPs Vorstand Feger es ausdrückte „allein nach wissenschaftlicher Notwendigkeit“ (1977: 15). Sie folgen vielmehr im Sinne eines situierten Wissens nach Donna Haraway (1988) den sozialen als auch politischen Interessen und Verortungen dominierender Akteure.

In feministischen Frauenzentren und -projekten hingegen waren Feministinnen die dominierenden Akteurinnen: Frauenpolitische Ambitionen und das Frausein an sich wurde zum Inklusions- statt zum Exklusionsfaktor. In diesem Umfeld wurden die Ziele der feministischen Therapie an denen der Bewegung ausgerichtet. Dies konnte professionelle feministische Therapeutinnen jedoch auch in diesem Kontext nur partiell legitimieren, da medizinischem sowie therapeutischem Expertentum in der Frauenbewegung mit großer Skepsis begegnet wurde. Doch ohne Expertise stießen aktivistische Beraterinnen in der Beratungspraxis schnell an Grenzen, wie das Beispiel der BIFF gezeigt hat. „Hilfe zur Selbsthilfe“ lautete das Zauberwort, das Therapie im feministischen Kontext möglich machte. Die Selbsthilfe galt – sofern sie unter Ausschluss von Männern stattfand – als am wenigsten von Hierarchien und Machtgefällen durchdrungenes therapeutisches Setting. Psychologinnen waren daran beteiligt, emanzipatorische Ideale der feministischen Frauengruppen in die therapeutische Arbeit mit Frauen zu übertragen. Dabei wurden in feministischen Kontexten nicht die Profession, sondern Anrufungen an die Frauensolidarität in den Vordergrund gestellt. Nichtsdestotrotz wurde die Idee einer feministischen Therapie von Anfang an auch von professionellen Bestrebungen getragen. Das Navigieren zwischen der feministischen Bewegung mit ihren radikalen Forderungen und Ansprüchen und den Anforderungen an professionelle Psychologinnen erforderte Geschick. Mit der Gründung eigener Projekte und der Organisation eines Kongresses eröffneten feministische Therapeutinnen sich die von Doderer und Kortendiek beschriebenen „emanzipatorische[n] Praxis- und Handlungsfelder“ (2008). Als Teil der im Entstehen begriffenen Frauengesundheitsbewegung adressierten sie die Frauenbefreiung auf psychischer Ebene. Es galt, die Entwicklung selbstständiger Frauenidentitäten zu fördern, die den „normalen“ Zumutungen des Patriarchats widerstehen können sollten, ohne sich davon „verrückt“ machen zu lassen.

## Footnotes

1 Wenn im folgenden Beitrag von Frauen ohne Asterisk (*) die Rede ist, folgt dies der historischen Selbstbezeichnung feministischer Akteurinnen der 1970er Jahren. Mit Asterisk werden Personengruppen gekennzeichnet, deren geschlechtliche Vielfalt abgebildet werden soll (z. B. Frauen*).

2 Die Bestände der Münchner Frauengesundheitsbewegung wurden vom Stadtarchiv München als sensibel eingestuft. Es gelten hohe Datenschutzbestimmungen, die das Nennen personenbezogener Angaben der Akteurinnen nicht erlauben.

3 Der Selbstbezeichnung der lokalen Akteurinnen aus Berlin (West) folgend wird im weiteren Text Berlin bzw. Berlinerinnen ohne den Zusatz West verwendet.

4 Gegenwärtig liegt der Frauenanteil im Psychologiestudium in Deutschland bei mehr als 75 Prozent (aerzteblatt.de 2022). In Baden-Württemberg waren 2019 gut 43 Prozent der Psychologieprofessorinnen weiblich (Statistisches Landesamt Baden-Württemberg 2021).

5 Der kurze Beitrag „OFP – nicht lebensfähig in der etablierten Psychologie?“ wurde pseudonymisiert verfasst (C. S. 1983).

6 OFP-Protokoll vom 01.02.1975, im Staatsarchiv München (StAM) KOE AMF: Organisation Frauen in der Psychologie OFP. Die im Staatsarchiv München archivierten und von der Frauenakademie München zusammengetragenen Archivalien der Münchner Frauengesundheitsbewegung waren zum Zeitpunkt der Archivrecherchen noch nicht mit Amtssignatur versehen, weshalb hier lediglich auf den Bestand (KOE AMF) und den Titel des Konvoluts (Organisation Frauen in der Psychologie OFP, kurz OFP) verwiesen wird.

7 OFP-Protokoll vom 25.09.1974: 1, in StAM KOE AMF: OFP.

8 Ebd.: 1.

9 Ebd.: 3.

10 Ebd.

11 Ebd.: 4.

12 Erfolgt zum Beispiel in *Psychologie Heute*. 1974 (3) 8.

13 *OFP-Info* Nr. 1 vom 11.11.1974, in StAM KOE-AMF: OFP.

14 OFP-Protokoll vom 08.10.[1974], in StAM KOE AMF: OFP.

15 OFP-Protokoll vom 01.–02.02.1975, in StAM KOE AMF: OFP.

16 Zudem fanden Arbeitsgruppen zu Geschlechtsstereotypen, zur „Arbeitsplatzsituation“ von Psychologinnen sowie zu Organisationsformen statt (ebd.: 2). Hiervon liegen jedoch im Bestand des Staatsarchivs München keine gesonderten Protokolle vor.

17 Protokoll der AG Forschung vom 01.02.1975: 1, in StAM KOE AMF: OFP.

18 Ebd.: 2.

19 Ebd.

20 Ebd.

21 Protokoll des AK Therapie vom 08.02.1975: 2, in StAM KOE AMF: OFP.

22 Ebd.: 2.

23 Ebd.: 1.

24 Ebd.

25 *OFP AKTUELL: Zeitung der „Organisation: Frauen in der Psychologie“ *1975 (1), StAM KOE AMF: OFP.

26 Ebd.: 2.

27 Ebd.: 3.

28 Ebd.

29 Die Broschüre wurde von der BIFF in West-Berlin im Selbstverlag produziert und herausgegeben und durch den Frauenverlag Frauenoffensive in West-Deutschland vertrieben. Das Original der hier dargestellten Abbildung befindet sich im Stichwort: Archiv der Frauen- und Lesbenbewegung, Gußhausstraße 20, 1040 Wien, Österreich: H BIF 6992

30 Das Münchner Modell der Psycho-Selbsthilfegruppen, undatiert, in StAM KOE AMF: Selbsthilfegruppen und Feministische Therapie (1970er Jahre).

31 OFP-Protokoll vom 25.09.1974: 1, in StAM KOE AMF: OFP.
